# Evaluating the Spatio-Temporal Factors that Structure Network Parameters of Plant-Herbivore Interactions

**DOI:** 10.1371/journal.pone.0110430

**Published:** 2014-10-23

**Authors:** Antonio López-Carretero, Cecilia Díaz-Castelazo, Karina Boege, Víctor Rico-Gray

**Affiliations:** 1 Instituto de Ecología A.C., Xalapa, Veracruz, México; 2 Instituto de Ecología, Universidad Nacional Autónoma de México, México D.F., México; 3 Instituto de Neuroetología, Universidad Veracruzana, Xalapa, Veracruz, México; Texas A&M University at Galveston, United States of America

## Abstract

Despite the dynamic nature of ecological interactions, most studies on species networks offer static representations of their structure, constraining our understanding of the ecological mechanisms involved in their spatio-temporal stability. This is the first study to evaluate plant-herbivore interaction networks on a small spatio-temporal scale. Specifically, we simultaneously assessed the effect of host plant availability, habitat complexity and seasonality on the structure of plant-herbivore networks in a coastal tropical ecosystem. Our results revealed that changes in the host plant community resulting from seasonality and habitat structure are reflected not only in the herbivore community, but also in the emergent properties (network parameters) of the plant-herbivore interaction network such as connectance, selectiveness and modularity. Habitat conditions and periods that are most stressful favored the presence of less selective and susceptible herbivore species, resulting in increased connectance within networks. In contrast, the high degree of selectivennes (i.e. interaction specialization) and modularity of the networks under less stressful conditions was promoted by the diversification in resource use by herbivores. By analyzing networks at a small spatio-temporal scale we identified the ecological factors structuring this network such as habitat complexity and seasonality. Our research offers new evidence on the role of abiotic and biotic factors in the variation of the properties of species interaction networks.

## Introduction

Approximately 40% of terrestrial biodiversity is involved in plant-herbivore species interaction networks [1]; these interactions are one of the main conduits for upward energy flow to higher trophic levels [2], and are mainly found in tropical ecosystems [3], [4]. Plant-herbivore interactions are considered antagonistic since the loss of tissue of the host plants caused by herbivore feeding may have detrimental consequences in their fitness or survival [5]. Unlike mutualistic interactions and networks, antagonistic networks tend to be more specialized and have an essentially modular structure [6], [7], characterized by cohesive groups of species that interact intensively (more than expected by chance) and maintain few interactions with other groups of species [8], [9].

Despite the advances in the identification and description of structural patterns of ecological networks, relatively little is known about the ecological and phylogenetic mechanisms that give rise to such patterns [10]. For plant-herbivore trophic networks it has been recently demonstrated that their structural attributes are influenced by species body size, local abundance, phylogeny [7], and by disturbance, since deforestation, habitat fragmentation and secondary plant succession all reduce the specialization and structural complexity of plant-herbivore networks owing to the resulting alteration of the food resources of herbivorous insects [11], [12], [13].

Furthermore, both seasonal changes in precipitation and structural complexity of plant communities are intimately related with changes in microclimate (e.g. temperature and humidity) and with the availability and quality of host plants for herbivores [14], [15], [16], [17]. Thus, both factors can have an important effect on the structure, composition and specialization of herbivorous insect communities [15], [18], [19]. In general, plant communities with high heterogeneity and species diversity offer a wide variety of food and habitats for herbivores and can reduce stressing factors such as temperature and humidity, which affect the performance of invertebrate herbivores [20], [21]. Such conditions promote a more diverse and specialized community of insects than in less structured plant communities with fewer host species and more stressful conditions [4], [21]. Although the effects of seasonality and variation in the quality of food resources on herbivore communities have been well documented for different ecosystems [14], [18], [22], the influence of these factors on interaction networks has not been evaluated.

Addressing the spatio-temporal assessment of ecological networks represents a new frontier in community ecology [23]. This approach can provide evidence of the influence of biotic and abiotic factors in the variation of species interaction networks [24], [25]. However, this information is particularly scarce for antagonistic interactions [7], [26]. In the present study, we analyzed plant-herbivore networks (lepidopteran larvae feeding on plants) at a small spatio-temporal scale, which has the advantage of identifying the ecological processes that influence the structure of such networks occurring within the same region (coastal habitats spaced as far as 0.4 ha) [27]. In particular we were interested in answering the following questions: i) What is the seasonal variation in the structural parameters of the plant-herbivore network in habitats that differ in their complexity? ii) How does resource availability vary across habitats and seasons? and, iii) Which is the simultaneous contribution of host availability, habitat structure and seasonality for the spatio-temporal structuring of this antagonistic network? In antagonistic systems coevolutionary processes will tend to reinforce constraints against grand generalists (only a small proportion of herbivorous animals are true generalists), thus compartments should often be apparent in such networks [9]. Since host use by phytophagous insects tend to specialization [28], compartmentation or modularity [8] and may be influenced by resource availability [6], [29] we hypothesized that: i) strong seasonal effects would be detectable at the structural parameters of plant-herbivore networks at the studied site, with higher selectiveness (network-level specialization) and modularity during the wet season and forest habitats, ii) host availability and interaction frequency would vary among habitats and temporal periods, thus we may expect iii) higher connectance, interaction evenness and lower selectiveness during dry season periods and open habitats.

This study is unique in the sense that, for the first time, the impacts of resource availability, habitat structural complexity and seasonality on the structure (parameters) of an antagonistic network were assessed.

## Methods

### Study site

Field work was carried out at La Mancha Coastal Research Center (Centro de Investigaciones Costeras La Mancha, CICOLMA) located on the coast of the state of Veracruz in Mexico (96°24′48″W, 19°40′33″N y 96°22′25″W, 19°31′49″N). Permit to perform the present research was issued by Instituto de Ecología, A.C. (INECOL), since CICOLMA is one of INECOL's field station and private conservation reserve. CICOLMA is a very small fraction of the 1336 Ramsar Site called “La Mancha el Llano” declared in 2005. Thus, the regulatory body concerned with protection of wildlife is given to INECOL by Mexican federal organisms in the matter and framed by The Ramsar Convention on Wetlands (intergovernmental treaty). The study site, the CICOLMA reserve area managed by INECOL covers an area of 70 ha, which includes several native vegetation types (including wetlands and dune vegetation), transformed forestry/agricultural sites, experimental areas and a field station. The major vegetation types here are tropical deciduous forest, tropical dry forest, sand dune scrub, mangrove forest, freshwater marsh and flooded deciduous forest [30], [31].

The climate of the study site is warm sub-humid and total annual precipitation ranges from 899 to 1829 mm, ca. 78% of the total annual precipitation falls during the rainy season (June - September). In this area, 837 plant species have been recorded, 50% of which are herbaceous plants and the rest are shrubs, trees and vines [30]. Currently, there is no list of lepidopteran species for CICOLMA. The field studies presented here did not involve endangered or protected species.

### Recording plant-herbivore interactions

The study was carried out at five sites with vegetation types representative of the plant communities in the study region and with differing degrees of structural complexity: 1) pioneer dune vegetation (PIO), 2) coastal dune scrub (DUN), 3) recently established tropical lowland subdeciduous forest (SFY) 4) tropical lowland subdeciduous forest in an advanced stage of succession (SFO), 5) tropical lowland flood forest with a wetland ecotone (FFW).

Structural complexity and vegetation strata differ specially between the open (PIO, DUN) and the forest habitats (SFY, SFO, FFW). The open habitats have moderate vegetation cover due to increased sand movement at the dunes, with dominant herbaceous stratum (although two strata are common at these habitats).These habitats are exposed to abiotic stressful conditions because of high temperatures, radiation, salt water influence and mechanical damage of vegetation during storms and hurricanes [32], [33]. In contrast, the forested habitats (SFY, SFO, FFW) are more stable and with milder conditions because of shade; vegetation structure at these habitats is more complex since three distinct arboreal strata occur here, the taller elements may exceed 20 m; the medium strata with trees of 6 to 15 m high, and finally, a well-developed understory [30].

At each of these vegetation types we placed 14 quadrats (4×4 m) where plant-herbivore interactions were recorded, along with the attributes of each plant community. In monthly censuses during the dry (March, April, May, June) and the wet/rainy (July, August, September, and October) season of 2012, we collected all folivorous caterpillar species (excluding leaf miners) associated with host herbs, lianas, shrubs and trees. In order to search for caterpillars on adult trees, a subsample of four branches was taken per individual tree. All caterpillars were recorded and taken to the laboratory where they were fed until pupated. No ethical approval was required for manipulating lepidopteran specimens, since after caterpillars pupated and adults emerged, only unidentified species (at larval stage) were mounted as adults for taxonomical identification (and only one individual of the unidentified species was mounted). Data underlying the findings of the present study are available within this manuscript, but lepidopteran specimens found at the present study are being processed for subsequent molecular analysis (Instituto de Ecología UNAM, México, by K. Boege) after which specimen identity will be publicly available in reference to this manuscript. Host plants were identified and deposited in the XAL Herbarium of the Instituto de Ecología, A.C. (INECOL, Xalapa, México).

### Structural network parameters

To assess spatio-temporal changes in the structural network parameters, for each of the five sampled vegetation types we constructed four matrices of the plant-caterpillar interactions throughout the dry and wet season: dry season 1 (DS1: March, April), dry season 2 (DS2: May, June), rainy season 1 (RS1: July, August) rainy season 2 (RS2: September, October). Previous work at the study site suggests that there is short-scale temporal variation in the structure of insect-plant –mutualistic- interaction networks derived from changes in abiotic properties (temperature and precipitation) [24]. In the present study, the monthly data of plant-herbivore interactions recorded at the field was fused bimestrially in order to have robust temporal information across vegetation types, since at some habitats, monthly census during dry season provide scarce interaction records; furthermore, these bimesters markedly reflect temporal biotic and abiotic changes at the study site: the beginning of the dry season period DS1 mark the end of cold fronts (“nortes”, see below), DS2 months corresponds to peak maximum temperatures, RS1 months corresponds to peak rainfall and RS2 correspond to the end of the rainy season. We did not make census from November to February because these months correspond to “nortes”, a season of winter cold fronts, characterized by strong winds and a considerable decrease in plant cover [34].

We obtained a total of 20 matrices of species interactions (that result from multiplying 5 vegetation types and 4 bimestrial censuses), which we refer to as sub-networks. We calculated the following structural parameters for each sub-network: connectance (C), Interaction evenness (IE), vulnerability (V), generality (G), modularity (M), number of modules (NM) and network specialization or selectiveness (selective use of the lower trophic level by the higher trophic level) of the entire bipartite network (H^2^), given that H^2^ describes to which extent observed interactions deviate from those that would be expected given the species marginal sums [27]. 1) Connectance was defined as the fraction of recorded interactions relative to the total number of possible interactions. 2) Interaction evenness indicates how homogeneously the plant-herbivore interactions are distributed throughout the network. It is calculated similarly to Shannon's Index, but does not take into account the absence of interactions (i.e., cells that contain a zero in the interaction matrix). Evenness approaches a value of 1 when the number of interactions between herbivore and host plant species is uniformly distributed. 3) Generality represents the proportion of herbivorous species to host species. 4) Vulnerability was calculated as the proportion of host species to herbivorous species. 5) The number of modules corresponds to the number of subgroups of the network that are not connected to other groups: modules are cohesive groups of highly connected nodes that are loosely connected to other nodes in the network [35], [36]. 6) Modularity is a measure of how host and herbivorous species tend to organize into subgroups of species that interact more frequently among themselves than with other members of the network. Because the degree of modularity determines how dense the connection between different groups of elements in an ecological system is, systems that largely differ in the degree of modularity often differ in their ecological and evolutionary [35]. This parameter was calculated with the M index (range: 0–1) in MODULAR [36] based on Newman and Girvan's algorithm. Its level of significance was calculated using Monte Carlo tests with 1000 randomizations. In order to carry out robust comparisons among sub-networks not affected by neither sampling effort nor network size, we provided a relative value of Modularity (Mr) estimated from the z-score values of the 1000 random replicates of the modularity analysis described above, as: Mr = M-Mz/Mz where M is the value of the real sub-network data matrix, while Mz refers to the average value of modularity of its random replicates, 7) Network specialization (H^2^) is based on the deviation between the observed and expected number of interactions in each network, assuming that all species interact with other species in proportion to their total observed frequency. Given that this metric is not affected by neither sampling effort nor network size, it is possible to make robust, reliable comparisons between different networks [27]. H^2^ values, ranging from 0 (low selectiveness) to 1 (high selectiveness). The network indices were calculated for each census and site using the BIPARTITE 3.0.1 package run in R [37].

### Attributes of the plant community

In each vegetation type and census, the following plant community attributes were quantified. As a measure of vegetation structure, total plant species richness (TPR) was registered, along with an estimation of canopy cover provided by all plant species (included host and non-host species) found in each quadrat (total plant cover  = TPC). As an indicator of food availability to herbivores, we measured plant cover of host species (i.e., exclusively those species in which caterpillars were found; host plant cover  = HPC). Species richness of host plants used by herbivorous lepidopterans (host plant richness  = HPR) was also registered. These variables were selected because they influence herbivore food availability, habitat structure and the abiotic factors that affect plant-herbivore interactions.

### Data analysis

#### Effect of vegetation type and seasonality on network parameters and plant community attributes

The effect of vegetation type and census on plant community attributes (TPR, TPC, HPC) and structural parameters of each network (C, G, V, IE, M, Mr, NM, H^2^) were evaluated using repeated measures ANOVAs, using the sub-network parameters as replicates for each vegetation type and census. Using a standard ANOVA in this case is not appropriate because it fails to model the correlation between the repeated measures (census and vegetation types): the data violate the ANOVA assumption of independence. Therefore the repeated measures ANOVA is suitable for analysis of auto correlated samples. To assess the influence of vegetation type, the model included census as the error term (within subject) and vegetation type as the explanatory variable (between subject). To evaluate the influence of census, the model included vegetation type as the error term (within subject) and census as the explanatory variable.

#### Spatio-temporal ordination of the sub-networks and their relation with plant community attributes

Plant-herbivore interactions were spatio-temporally analyzed at 20 sub-networks (i.e. 20 frequency matrices). Structural network parameters were subsequently estimated for each sub-network. In order to describe the dissimilarity among these matrices, we performed a NMDS analysis (Non-metric Multi-Dimensional Scaling). The resulting NMDS ordination (biplot) shows the structural parameters of all sub-networks: when sub-networks are close to a specific parameter, these have small dissimilarities in their values for that parameter. This is one of the most effective methods for the ordination of ecological data and the identification of underlying gradients, because it does not assume a linear relationship among the variables [38]. Because this analysis offers more than one solution, we carried out an iterative process to find the model with smallest stress value using the metaMDS function in the MASS routine run in R [39].

To evaluate if spatio-temporal dissimilarity among sub-network parameters was related to plant community attributes, we analyzed the effect of TPR, TPC, HPC (continuous variables) (see “Attributes of the plant community” within this section) on the ordination of plant-herbivore sub-networks using the envfit function from the VEGAN library of the statistical package R [39]. This function fits the vectors or continuous values of the environmental variables (TPR, TPC, HPC) to the NMDS ordination axes, generating a measure of correlation (r) and a significance value based on the probability that 1000 random permutations of environmental variables would have a better fit than the real environmental variables.

To test the null hypothesis of a similar composition of herbivore and host plants species among vegetation types and sampling censuses, we used a PERMANOVA (Permutational Multivariate Analysis of Variance), and the Bray-Curtis Index [40]. The PERMANOVA was performed using the *adonis* function of the VEGAN package run in R. This function runs a multivariate analysis using the distance between matrices (dissimilarities between objects or sampling sites), and the probability of significance P is obtained through permutations [40]. For these analyses, we used the following groups: season (censuses of the dry season vs. censuses of the rainy season) and structural complexity of the habitat (open habitats vs. forest).

To understand if spatio-temporal changes in herbivore species composition (ordination dissimilarity) could affect network structure, we analyzed the effect of parameters C, H^2^, M y NM (quantitative variables or vectors) over the ordination (biplot) of an NMDS analysis of the community of lepidopteran herbivorous larvae found foraging on plants at the 20 sub-networks using the *envfit* function from the VEGAN package of the statistical software R. This function provides a correlation measure and is statistical significance (after comparing correlation values with 1000 random permutations).

## Results

### Effect of vegetation type and seasonality on network structure and plant community attributes

We recorded 654 larvae belonging to 176 morphospecies of Lepidoptera and associated with 56 host plant species. During the dry season there were 237 larvae belonging to 41 morphospecies of Lepidoptera feeding on 33 species of plants, whereas during the rainy season there were 417 larvae belonging to 66 species of Lepidoptera feeding on 47 species of host plants. The composition of the herbivore and host plant communities differed significantly between the studied open and forested habitats (PERMANOVA, herbivores: Pseudo-F = 2.667; P = 0.004, plants: Pseudo-F = 4.043; P = 0.004). For both herbivores (lepidopteran larvae) and hosts plants, species turnover was higher between groups (vegetation types) than within them (Tukey test, herbivores: P = 0.787, plants: P = 0.719). The mean values of C, M, NM and H^2^ of the network varied significantly across censuses and among vegetation types (Table 1A). The network parameter V changed across time, while G and IE were not different between vegetation types or throughout the year (Tables 1A).

**Table 1:** Results of the repeated measures analysis of variance (ANOVA) for the effect of census and vegetation type on structural network parameters (A) and the plant community attributes (B).

(A) Network parameters	*DF*	*SS*	*F*	*P*
**Generality (G)**				
Census	3	0.005	0.626	0.614
Vegetation type	4	0.004	0.363	0.831
**Vulnerability (V)**				
Census	3	1.961	4.167	***0.033***
Vegetation type	4	0.645	1.028	0.434
**I. Evenness (IE)**				
Census	3	0.034	1.808	0.203
Vegetation type	4	0.461	1.803	0.198
**Connectance (C)**				
Census	3	0.099	29.218	*<0.001*
Vegetation type	4	0.066	14.698	*<0.001*
**Modularity (M)**				
Census	3	0.125	11.568	***0.001***
Vegetation type	4	0.128	8.896	***0.009***
**No. Modules (NM)**				
Census	3	0.125	11.568	***0.001***
Vegetation type	4	0.128	8.896	***<0.001***
**Network Specialization (H_2_)**				
Census	3	5.842	22.023	***<0.001***
Vegetation type	4	1.483	4.195	***0.026***

(B) Attributes of the plant community				
**Total Plant Cover (TPC)**				
Census	3	642.717	0.912	0.466
Vegetation type	4	1895.716	2.011	0.161
**Total Plant Richness (TPR)**				
Census	3	48.133	1.079	0.397
Vegetation type	4	1678.228	28.231	***<0.001***
**Host Plant Cover (HPC)**				
Census	3	0.342	10.462	***0.001***
Vegetation type	4	0.892	20.456	***<0.001***
**Host Plant Richness (HPR)**				
Census	3	222.64	11.478	***0.001***
Vegetation type	4	176.95	6.776	***0.005***

Significant values of P (≤0.05) are in bold and italics.

**Figure 1:**
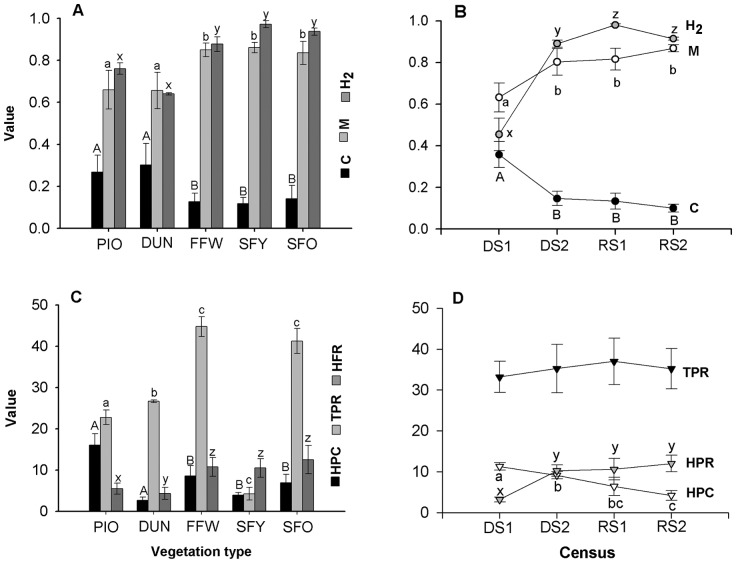
Vegetation type and seasonality effect on network parameters and plant community attributes. Vegetation types (PIO: pioneer dune vegetation, DUN: coastal dune scrub, FFW: flood forest with a wetland ecotone, SFY: recently established tropical lowland subdeciduous forest, SFO: tropical lowland subdeciduous forest in an advanced stage of succession). Census (DS1: Dry season 1, DS2: Dry season 2, RS1: Rainy season 1, RS2: Rainy season 2). Network parameters (H^2^: network specialization, M: Modularity, C: Connectance). Plant community attributes (TPR: Total plant richness, HPR: Host plant richness, HPC: Host plant cover). (A) Network parameters by vegetation type. (B) Network parameters by census. (C) Plant community attributes by vegetation type. (D) Plant community attributes by census. Comparisons are valid only within each network parameter (panels A and B, for vegetation types and census, respectively) and within each plant community attribute (panels C and D, for vegetation types and census, respectively). Bars indicate standard error of the mean. Different letters represent significant differences resulting from post-hoc test (P<0.05).

Post hoc tests indicated that the values for M, Mr, NM and H^2^ of the forest habitats (SFY, SFO, FFW) were significantly higher than those with open vegetation (PIO, DUN) (Figure 1A, Table 2). Additionally, the networks of open habitats had the highest C, with values almost twice than those of forest habitats. The network indices did not vary significantly among the three types of forest vegetation, or between the two types of open vegetation (Figure 1A).

**Table 2:** Relative modularity (Mr) based in z-score values estimated for the vegetation types and censuses (mean ±SD) in plant-herbivore sub-networks.

Vegetation type	Modularity (Mr)
PIO	1.887±0.303^a^
DUN	1.907±0.194^a^
FFW	2.611±0.111^b^
SFY	2.696±0.191^b^
SFO	2.669±0.140^b^
**Census**	
DS1	2.049±0.227^a^
DS2	2.325±0.322^ab^
RS1	2.440±0.242^bc^
RS2	2.686±0.108^c^

Different letters among vegetation types and censuses represent significant differences in post-hoc test at P≤0.05. See Methods section for vegetation types and censuses abbreviations.

The first census of the dry season had the highest connectance of the entire study, with values twice as high as during the rest of the year (Figure 1B). In contrast, species networks of the last three censuses were characterized by high M, Mr (Table 2), NM and H^2^ values that were twice as much of those estimated for the networks of the first census. None of these indices were different among the last three censuses (Figure 1B). It is important to notice that all sub-networks (but one) were significantly modular (Table 2).

The HPC and HPR varied significantly among vegetation types and across censuses, while total floristic richness differed only among vegetation types (Table 1B, Figure 1). The TPC did not differ statistically between vegetation types or censuses. In particular HPC in PIO was two times greater than in FFW and SFO and three times greater than in DUN and SFY. In contrast, the DUN had the lowest host plant cover of all the vegetation types studied, even with respect to the PIO (Figure 1C). The TPR and HPR in the forest habitats were twice than in open habitats (Figure 1C).

During the dry season, HPC was two times that of the wet season. Although TPR did not differ statistically across time, the HPR was about three times lower during DS1 relative to the rest of the year (Table 1B, Figure 1D). By comparing TPR and HPR values, we found that in all vegetation types and throughout all censuses, herbivores used approximately one quarter of the available plant species (Figure 1 C–D).

### Spatio-temporal ordination of the sub-networks and their relation with plant community attributes

**Figure 2:**
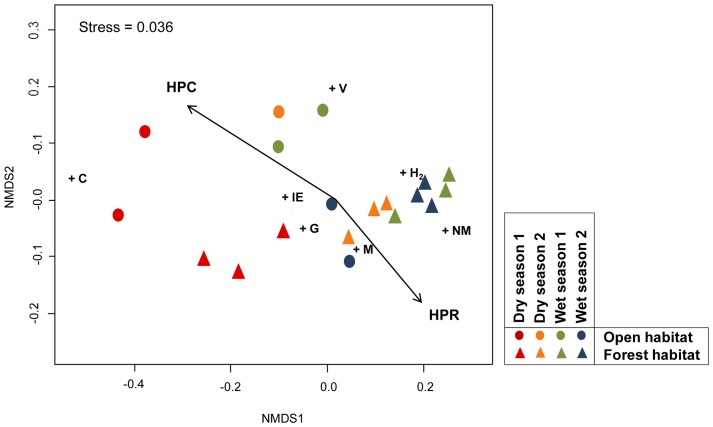
NMDS ordination of network parameters showing the spatio-temporal variation of plant-herbivore sub-networks. The plus signs (+) indicate the network parameters upon which the sub-network ordination was performed (H^2^: network specialization, M: Modularity, C: Connectance, IE: interaction evenness, V: vulnerability, G: generality). The vectors on the ordination represent the gradient in HPR (Host plant richness) and HPC (Host plant cover) for all sub-networks. The arrow points to the direction in which the linear change in HPR and HPC was strongest, and the length of the arrow is proportional to the correlation between these variables and the sub-network ordination. Arrows were plotted only for variables with significance of P≤0.05. Factors fit in the ordination (season and habitat type) are not included. For habitats abbreviations see Figure 1.

In the NMDS of plant-herbivore sub-networks, axis 1 was associated with the slope defined by seasonality (Figure 2). During the first census of the dry season the sub-networks clustered independently of the vegetation type because of their high C values. In addition sub-networks exhibited a clear separation between the dry and wet seasons, characterized by their high degree of H^2^, M and NM (Figure 2). The sub-networks in the open vegetation habitats were defined by high values of C during the dry season. However, during the rainy season the sub-networks in these habitats were characterized by high values of IE, G and M (Figure 2). During the dry season, forest vegetation sub-networks had high values of IE and V, while the sub-networks of the rainy season had similarly high values of H^2^, M and NM (Figure 2).

Some attributes of plant communities influenced the structure of plant-herbivore sub-networks: vector TPR (r = 0.485, P = 0.003) and vector HPC (r = 0.274, P = 0.046). The correlation of HPC was greater for the ordination of the sub-networks in habitats with open vegetation and during the dry season (Figure 2). TPR had the highest degree of correlation with sub-network ordination, and its correlation was stronger for the ordination of the sub-networks of the forest vegetation types and rainy season (Figure 2).

**Figure 3:**
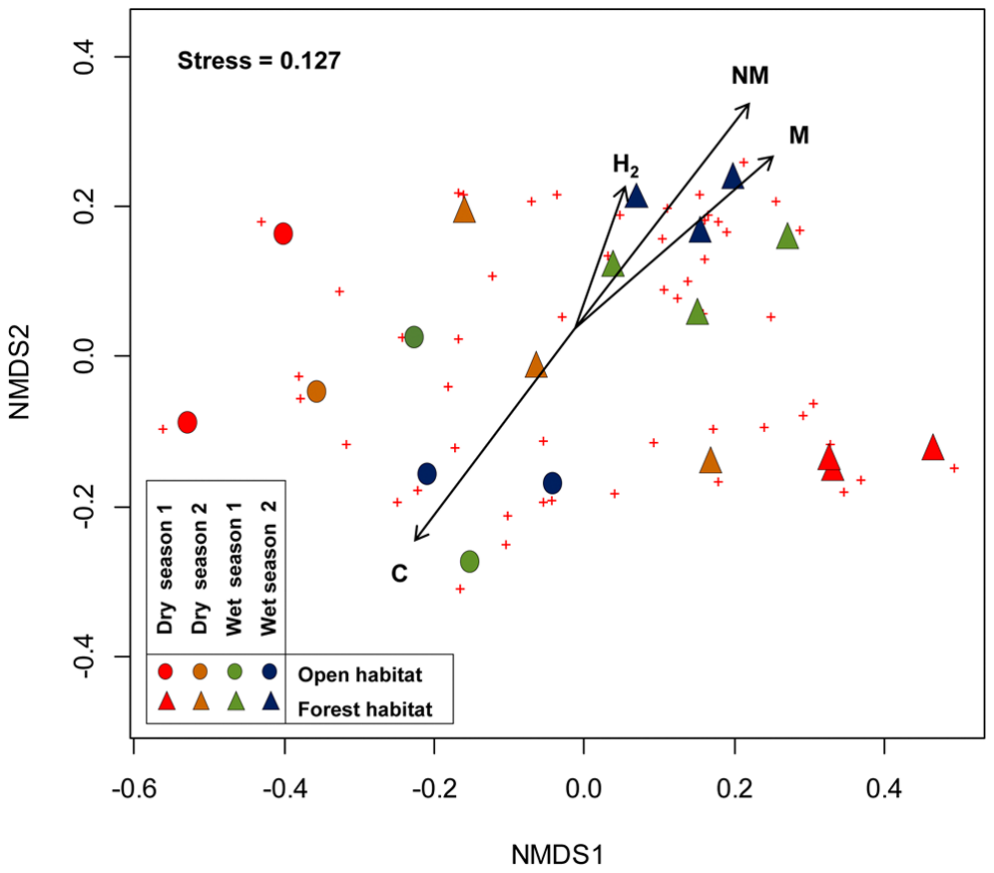
NMDS ordination of lepidopteran herbivore species composition showing the spatio-temporal variation in sub-networks. The plus signs (+) indicate different herbivore species upon which the sub-network ordination was performed. The vectors on the ordination represent the gradient in network parameter values across all sub-networks (for habitats abbreviations see Figure 1): H^2^: network specialization, M: Modularity, NM: Number of modules, C: Connectance. The arrow points to the direction in which the linear change in network parameters was strongest, and the length of the arrow is proportional to the correlation between these variables and the ordination of herbivore species composition.

The NMDS exploring the relation among sub-network parameters and herbivore species composition showed an important spatio-temporal turnover of lepidopteran herbivore species (Figure 3). Independently of the bimester (seasonality), we found a clear dissimilarity among lepidopteran species foraging in plants at open habitats (PIO, DUN) and forest habitats (FFW, SFY, SFO). Vector fitting at NMDS showed that parameters NM, M and C were significantly correlated to dissimilarity in lepidopteran species composition (r = 0.464, P = 0.007; r = 0.389, P = 0.033; r = 0.364, P = 0.024, respectively). The correlation of M and NM was greater for the ordination of the sub-networks in habitats with forest vegetation, while the highest correlation of C occurred at open habitats (Figure 3). Thus, there are differences in lepidopteran species composition between sites that transcend to network structure (parameters): changes in lepidopteran species composition affects network general topology and connectance, but not specialization (selectiveness) (r = 0.236, P = 0.123).

## Discussion

It has recently been demonstrated that the structure of plant-herbivore interaction networks is influenced by ecological, phylogenetic [7] and anthropogenic attributes [12], [13], [41]. However, although the patterns of plant-herbivore interactions can be influenced by finer and more immediate environmental factors such as temporal variation in the quality and availability of food [18], [42] or habitat structure [4], [43], the impact of these factors on the properties of species interaction networks had not been evaluated.

Our results show for the first time how the structural parameters of plant-herbivore networks are spatially and temporally dynamic, influenced by seasonality, habitat complexity and food resource availability. The spatio-temporal changes in host plant communities resulting from abiotic factors (climate and micro environmental variation) were not only reflected in the species composition of herbivore community as previously reported [14], but also in the emergent properties of the antagonistic network such as its connectance, specialization (selectiveness) and modularity.

In contrast with mutualistic plant-insect networks, which maintain their structural stability over time [44], [45], [46], [47], antagonistic networks (i.e., plant-herbivore) tend to maintain their modular structure [4], [8]. Indeed, in this study plant-herbivore networks were characterized by many specialist interactions and a marked compartmentalization, as has been reported for other plant-insect herbivore networks [6], [7], [8], [48]. To our knowledge this is the first study to report structural seasonal and spatial changes in a plant-herbivore interaction network. Our interaction sampling is robust; since our study habitats are relatively close to each other (spaced as far as 0.4 ha) and the area of each habitat is reduced, we could established permanent quadrats (n = 14) that covered most environmental gradient; thus we have a considerably good sampling effort in reference to the interactions studied. In general, in order to estimate sampling completeness within interaction networks, the number (richness) of interactions accumulated as sampling effort increased is frequently used [49]. Since in our study the assemblage of lepidopteran herbivores showed a high proportion of rare species, the result is similar to other tropical communities of herbivorous insects [3], [50] where asymptotic interaction richness is not reached [15], [29]. This non-asymptotic pattern of accumulation curves occurs as well for lepidopteran species richness hosted even by plant species of the same genus or family [3], [51].

Although connectance is a simple parameter sensitive to network size [52], the inverse relation trend generally found between connectance and network size stabilizes after a “threshold” above 50 species conforming the network [53]. Since at the present study our smaller sub-networks have much more than 50 nodes, we consider that connectance can be comparatively used to describe spatio-temporal changes in plant-herbivore interaction “saturation”. In general, selectiveness (H^2^) metric is not affected by neither sampling effort nor network size [27], and the relative modularity (Mr) provided by the z-score values of “M” metric make our spatio-temporal comparisons robust. In our study, temporal variation in the structure of plant-herbivore networks was associated with changes in climatic factors (e.g., precipitation and temperature) during the transition from the dry to the rainy season, and was accompanied by a decrease in connectance (lower interaction saturation in the community) and an increase in the selectiveness/modularity of networks. These temporal changes in the configuration of species networks could be associated with a high susceptibility of monophagous or oligophagous lepidopteran herbivores that need to synchronize their life cycles to plant phenology [14], [18], [22]. Given that at the present study, no traits of plant species are provided, the H^2^ pattern among sub-networks suggest that, independently of the number of species constituting each sub-network, there is higher selectiveness of lepidopteran larvae (not necessarily trophic specialization) during the rainy season when offer of plant species (and foliage availability) is higher; this pattern may reflect changes in host choice by adult females, as a function of temporal changes in plant community [54], [55], [56]. Further studies including nutritional or defensive traits of plants used by herbivores at these networks or phylogenetic signaling of these interactions would shed light on the process generating these patterns.

The ordination of the most specialized and highly modular sub-networks was highly correlated with floristic richness of forest habitats and the rainy season. The high degree of specialization and modularity of the networks in the richest floristic contexts could be related to a greater diversification by the herbivores in their use of plant resources: a greater availability of niches at the lower trophic level is likely to promote a greater niche differentiation (and therefore greater specialization) in the higher trophic level. The forest sites (tropical lowland subdeciduous forest) were not only floristically the richest of the studied sites, but also offered more canopy cover from trees and shrubs, as well as more plants with different life forms [30]. Greater plant richness and structural complexity of the vegetation provides a wide variety of microhabitats and food that can result in lower competition by food resources among herbivores [4], [57]. In turn, this could translate into greater specialization and modularity in the network patterns. There are two possible reasons that might promote specialization of plant-herbivore networks in more floristically diverse contexts: i) the advantages conferred by specialist foraging behavior in the face of the difficulties herbivores have choosing suitable hosts [58] and/or ii) specialization in host species that provide herbivores enemy-free space in more structurally complex habitats [59], [60]. These avenues remain unexplored in a plant-herbivore network context.

The highest sub-network connectance was found under the most stressful abiotic conditions: Open vegetation and the dry season create conditions that could cause greater water stress in plants as a result of high temperatures and solar radiation. In turn, this could affect their nutrition (water and nitrogen content) and a defense (toughness and phenolic compounds content) attributes rendering the plants unsuitable for many lepidopteran herbivores [14], [18], [19], [42], [61]. For example, the coastal dune scrub and pioneer dune vegetation have the most extreme temperatures (up to 60°C in the scrub) and solar radiation [31], [32] of all our study sites. Moreover, the pioneer dune vegetation is subject to continual disturbance (burial under moving sand, strong winds with a high saline concentration, abrasion from blown sand, flooding and mechanical damage caused by tropical storms and hurricanes) [33], which makes host plants in these habitats highly unpredictable resource for herbivores [62]. In this context, both the unpredictable availability and low nutritional quality of the host plants in open habitats and present during the dry season can impose strong selective pressure on the herbivores [42], favoring species that are less specialized and less susceptible to variations in host resources [63], [64], [65]. In fact, in open vegetation habitats and during the dry season we found a predominance of herbivore species with relatively low host specificity. A generalist habit could make these species less susceptible to fluctuations in resource availability and quality under stressing conditions given that they have more food options.

At the present study most generalist herbivores were associated with host plants with large plant cover, mainly pioneer species adapted to stressful natural habitat conditions such as *Chamaecrista chamaecristoides, Crotalaria incana* and *Porophyllum punctatum* [33], [66]. These host species may have provided protection and food under the stressful abiotic conditions and reduced nutritional quality of the plants. Greater plant cover is a structural attribute that provides greater food availability to herbivores that can take advantage of this cover, and would allow them to compensate for the decrease in leaf nutrition quality [67], [68], and reduce larval desiccation under the high temperatures that characterize these environments.

In antagonistic networks, connectance - whether promoted by the range of host plants eaten by herbivores or by resource availability- can mitigate the loss of species when environmental conditions are unstable or stressful [12]. Therefore, the increase in connectance found in our study may have buffered the negative effects of natural disturbance (in stressful habitats and conditions) in plant-herbivore networks. Furthermore, the high network specialization was a structural attribute characteristic of forest habitats with stable host plant population size, compared with open habitats where specialist insects exhibited less spatio-temporal variation [69]. These results are evidence that the structure of antagonistic networks, just as it occurs in mutualistic networks, tends to reduce species vulnerability to extinction, which promotes community stability [70], [71].

In the studied plant-herbivore sub-networks, higher connectance was detected during the dry season in the pioneer habitat, and this seems similar to the changes that other trophic networks undergo when subjected to anthropic disturbances. The way in which the loss of arboreal cover reduced network complexity has been reported as a process of spatio-temporal homogenization of species and their interactions [12], [41], [72]. Moreover, both habitat fragmentation and early secondary succession can decrease the size of the network, resulting in systems that are more connected and less modular owing to the possible loss of specialists [12], [13], [72], [73]. At the study site, the pioneer habitat and the dry season may, represent a significant decrease in resource availability, particularly in the abundance of rare plants and their specialist herbivores. Additionally, the lack of arboreal cover in this habitat increases stressful abiotic conditions for herbivore larvae.

An important property of our plant-herbivore network was the spatio-temporal prevalence of vulnerability and interaction evenness. Regardless of habitat type or season, the ratio of herbivore to plant species was the lowest and no dominant interactions were recorded. Our results are similar to those of other trophic networks for habitats with differing degrees of fragmentation and where there was no evidence of variation in the structural attributes of the network [11]. In contrast, in parasitoid-herbivore trophic networks the spatio-temporal variation in interaction evenness is greater where deforested habitats are dominated by a reduced number of interactions [72]. In our study, the spatio-temporal prevalence of these indices could be a consequence of the high specialization plant-herbivore interactions [12], since the great majority of lepidopteran herbivores in tropical ecosystems are rare species that are low in abundance [14], even in disturbed tropical habitats [74]. Low vulnerability could also be a consequence of the loss of higher trophic levels as occurs in very small habitat fragments [12] [75].

In recent years, there have been efforts to assess the spatio-temporal variation of ecological networks [44], [45], [47], [76]. To our knowledge, this is the first study to address how plant-herbivore trophic networks vary in space and time at small scales, and to evaluate simultaneously the role of habitat complexity, resource availability and seasonality in such variation. This approach opens a new frontier in the study of plant-herbivore interactions in an effort to understand the processes that drive the modular and specialized patterns observed in antagonistic interaction networks.
